# Supplementary material for: To switch or not to switch – a machine learning approach for ferroelectricity
Source: Nanoscale Adv. 2020 Apr 15;2(5):2063–72. doi: 10.1039/c9na00731h (PMC9419588; doi:10.1039/c9na00731h)
Supplement: NA-002-C9NA00731H-s001 [file NA-002-C9NA00731H-s001.pdf]

Electronic Supplementary Material (ESI) for Nanoscale Advances.
This journal is © The Royal Society of Chemistry 2020

SUPPLEMENTARY INFORMATION

To Switch or Not to Switch – a Machine Learning Approach for Ferroelectricity

Sabine M. Neumayer[1], Stephen Jesse[1], Gabriel Velarde[2], Andrei L. Kholkin[3], Ivan Kravchenko[1],

Lane W. Martin[2], Nina Balke[1], Peter Maksymovych[1*]

[1] Center for Nanophase Materials Sciences, Oak Ridge National Laboratory, Oak Ridge, TN 37831, USA
[2] Department of Materials Science and Engineering, University of California, Berkeley, Berkeley, CA 94720, USA & Materials Sciences Division, Lawrence Berkeley National Laboratory, Berkeley, CA 94720, USA
[3] Department of Physics & CICECO -Aveiro Institute of Materials, University of Aveiro, Aveiro, Portugal & School of Natural Sciences and Mathematics, Ural Federal University, Ekaterinburg, Russia

*maksymovychp@ornl.gov

Section I

Experimental cKPFM data and interpolation extracted from cKPFM maps shown in Figure 3 for (a) PZT and (b) $aHfO_2$ between data points. Interpolation was performed using the scikit-image transform function.[40]

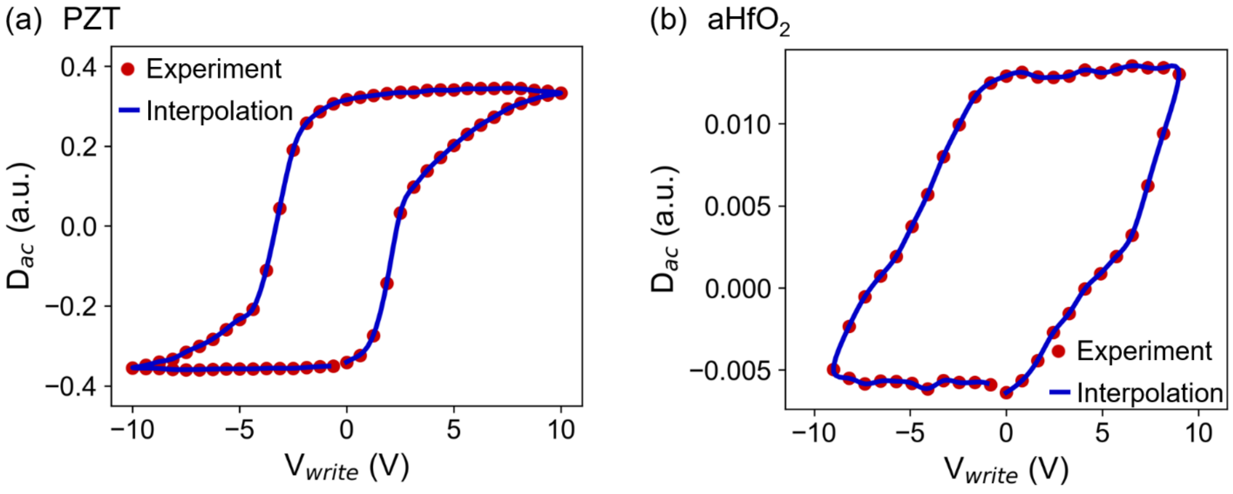

Figure S1: Experimental and interpolated response data measured (a) on PZT as a function of V_{write} and (b) on $aHfO_2$. Data were extracted from the cKPFM maps shown in Figure 3 at $_{Vread}$ = 0 V.

Section II

Maps of experimental and interpolated cKPFM data and their x- and y- gradients shown for different Vread and Vwrite amplitudes for PZT (Figure S2) and $aHfO_2$ (Figure S3).

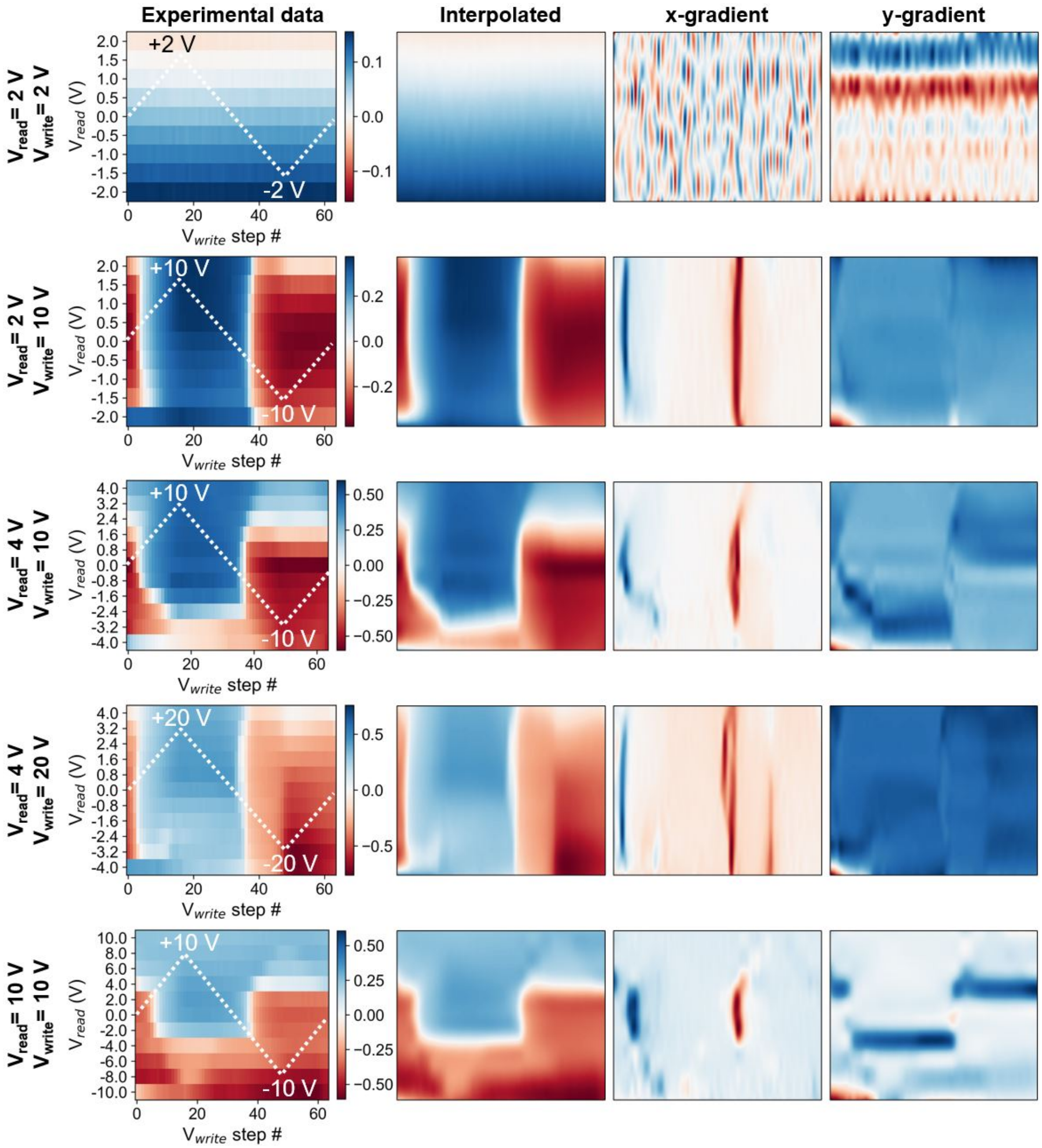

Figure S2: Maps of experimental cKPFM data acquired on PZT, interpolated data and the gradients in x- and y-direction calculated from interpolated maps. The V_{read} and V_{write} ratios vary according to the labels on the left. The first row shows response to sub-coercive read and write voltages.

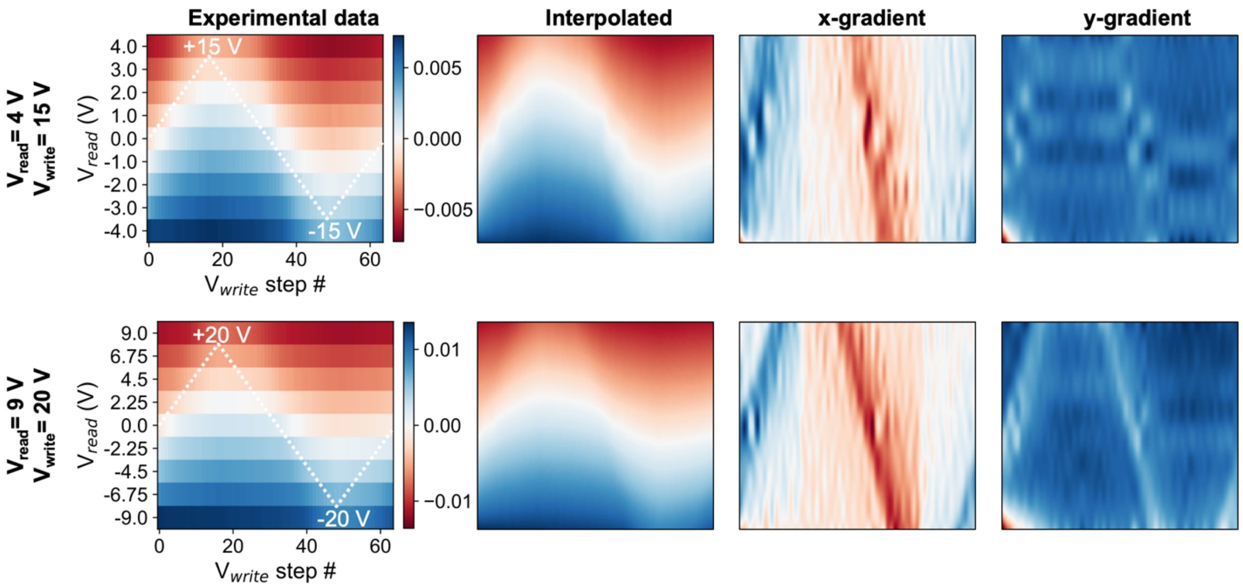

Figure S3: Maps of experimental cKPFM data acquired on $aHfO_2$, interpolated data and the gradients in x- and y-direction calculated from interpolated maps. The V_{read} and V_{write} amplitudes vary according to the labels on the left.

Section III

A multilayer perceptron ANN was trained on cKPFM maps within the data set shown in Figure 5(a), where 100 maps were acquired at 7 different temperature steps on PLZT across the ferroelectric – relaxor phase transition. Subsequently, the ANN was used to identify the temperature at which test data sets were measured.

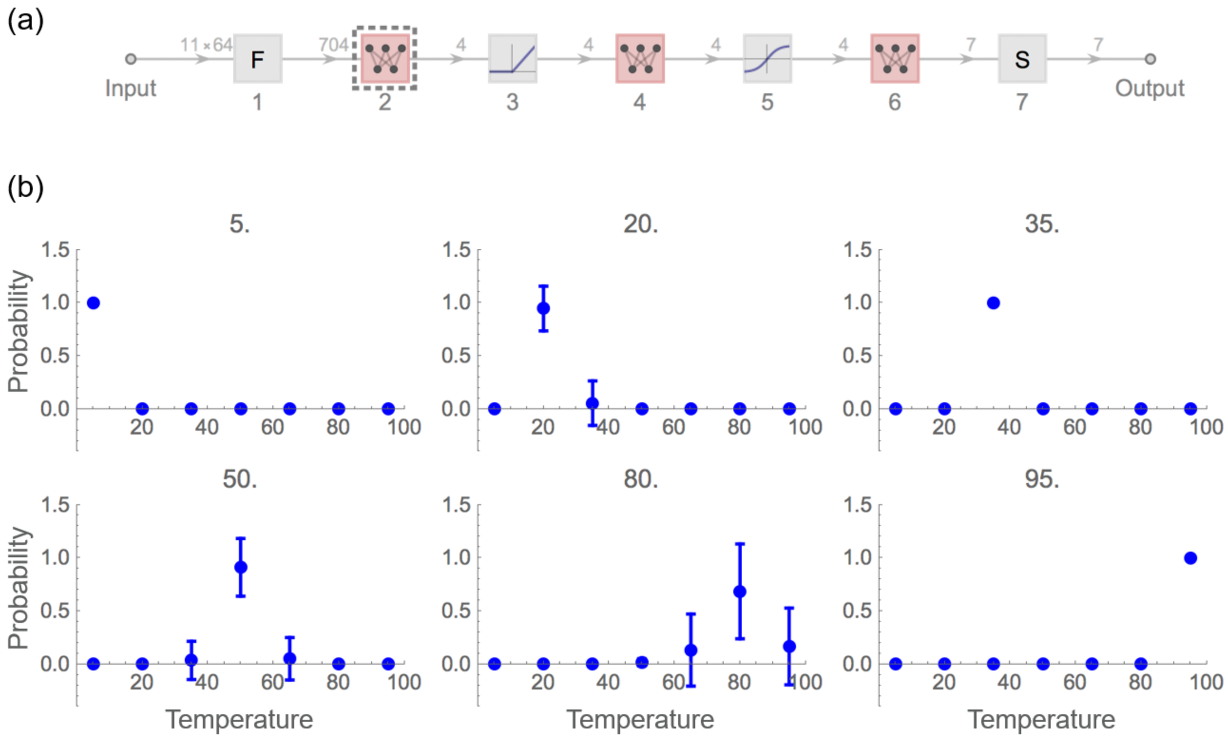

Figure S4: (a) Graph of linear multi-perceptron neural network used to predict temperatures based on the structure of the 2D response function. Layer structure of the network is as follows: (1) flattening image into a 704-dimensional vector; (2) mapping into 4 layer perceptron; (3) rectifying activation; (4) another mapping into 4 layer perceptron; (5) sigmoid activation; (6) mapping into 7 layer perceptron; (7) softmax normalization layer for normalization into class labels (each corresponding to specific temperature); (b) shows the average performance of the network to predict a specific temperature (labeled at the top) obtained by applying the network to validation data-set. (x – axis in each plot is the temperature in C; y-axis is the probability of predicted temperature)
